# Posterior wall thickness of the confluent inferior pulmonary veins measured by left atrial intracardiac echocardiography: implications for catheter ablation

**DOI:** 10.1007/s10840-023-01613-w

**Published:** 2023-07-25

**Authors:** Kodai Negishi, Ken Okumura, Fumitaka Onishi, Akino Yoshimura, Hideharu Okamatsu, Takuo Tsurugi, Yasuaki Tanaka, Yoshiro Sakai, Koichi Nakao, Tomohiro Sakamoto, Junjiro Koyama, Hirofumi Tomita

**Affiliations:** 1 Division of Cardiology, Saiseikai Kumamoto Hospital Cardiovascular Center, 5-3-1 Chikami Minami-Ku, Kumamoto, 861-4193 Japan; 2 Department of Cardiology and Nephrology, Hirosaki University Graduate School of Medicine, Hirosaki, Japan

**Keywords:** Confluent inferior pulmonary veins, Atrial fibrillation, Intracardiac echocardiography, Catheter ablation

## Abstract

**Background:**

Confluent inferior pulmonary veins (CIPV) is a rare anatomical variant. There is few evidence in the literature regarding anatomic landmarks consideration to guide radiofrequency application in avoiding complications in the esophagus in CIPV cases.

**Methods:**

Of 986 consecutive patients undergoing atrial fibrillation (AF) ablation from July 2020 to June 2022, seven (0.7%) had CIPV with a common trunk connecting to the LA diagnosed by 3-dimensional contrast-enhanced computed tomography. Using intracardiac echocardiography (ICE) performed from the left atrium (LA), we measured the posterior wall thickness (PWT) of the CIPV adjacent to the esophagus and compared the measurement with the LA posterior wall thickness (LAPWT) at the left inferior PV level of 25 controls without CIPV. For ablation in CIPV patients, each superior PV was individually isolated, and box isolation of CIPV without ablating the CIPV posterior wall was added (tri-circle ablation technique).

**Results:**

The CIPV PWT was 0.7 ± 0.1 mm, while non-CIPV LAPWT was 2.0 ± 0.4 mm (*P* < 0.001). In the CIPV group, upper and lower portions of the CIPV were both apart from the esophagus (mean distances, 6.7 ± 3.4 mm and 7.9 ± 2.7 mm, respectively). Individual superior PV isolation and box CIPV isolation resulted in complete isolation of all PVs, with no complications. All CIPV patients except one remained AF recurrence-free for 376 ± 52 days.

**Conclusions:**

Although CIPV frequency is low, CIPV PWT is very thin and special care is needed during ablation. A “tri-circle” ablation strategy avoids ablating in the thinnest portion of the posterior wall. Further studies are warranted to assess the safety.

**Supplementary Information:**

The online version contains supplementary material available at 10.1007/s10840-023-01613-w.

## Introduction

Pulmonary vein (PV) isolation (PVI) for atrial fibrillation (AF) is a well-established, effective treatment for preventing AF recurrence [1]. Currently, the broadly accepted ablation strategy for PVI is circumferential PVI, which creates a linear radiofrequency (RF) lesion on the ipsilateral pulmonary vein antrum [2].

The widespread use of AF ablation and advances in imaging technologies have increased awareness of variations in PV anatomy. Among the PV anatomic variations, confluent or common ostium inferior pulmonary veins (CIPV) is rare (0.9–1.5%) [3–8]. Of note, the morphologic characteristics of CIPV may make the PVI procedure difficult. Specifically, due to the proximity of the CIPV to the esophagus, the usual circumferential PVI procedure may be associated with a higher risk of esophageal injury, including an atrio-esophageal fistula, compared to the procedure for non-CIPV cases. Alternatively, tri-circle or box isolation ablation line strategies have been proposed for CIPV cases in a few case report series [7, 8].

Intracardiac echocardiography (ICE) with a Carto SoundStar® ultrasound catheter (Biosense Webster, Diamond Bar, CA, USA) was used for transseptal puncture during the AF ablation procedure. ICE with a Carto SoundStar® ultrasound catheter was also used for creating the left atrium (LA) 3-dimensional (3D) image from the right atrium, and this LA 3D image was used for integration with the LA 3D computed tomography (CT) image with the CartoMerge™ module (Biosense Webster) with high accuracy [9]. In our laboratory, by inserting the Carto SoundStar® catheter into the LA cavity through the long sheath advanced to the LA, we directly visualized the LA posterior wall and the esophagus from the LA in every AF ablation case. This method allowed us to create the 3D esophagus image adjacent to the LA posterior wall in a real-time fashion during the AF ablation procedure and to accurately assess the distance between the esophagus and the LA posterior wall [10]. Similarly, it was possible to assess the relationship between the esophagus and CIPV, which eventually resulted in risk assessment of esophageal injury. With the use of the ICE images of the LA posterior wall and esophagus, we measured the posterior wall thickness (PWT) of the CIPV and compared the PWT of the CIPV with the LA posterior wall thickness (LAPWT) in non-CIPV cases. We further studied the effectiveness of the box isolation line around the CIPV (tri-circle technique) for complete PVI. This is the first study demonstrating CIPV thickness compared to the LAPWT of patients without CIPV.

## Methods

### Study patients

**Fig. 1:**
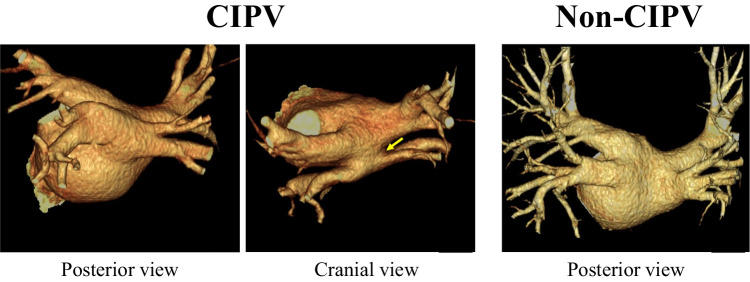
3-dimensional computed tomographic images of the left atrium (LA) of confluent inferior pulmonary veins (CIPV) and non-CIPV cases. In CIPV cases, the left and right inferior pulmonary veins had continuity behind the LA posterior wall with a common trunk (yellow arrow) connecting to the LA

Of the consecutive 986 patients undergoing their first AF ablation from July 2020 to June 2022, 7 had CIPV that was diagnosed by the LA image of 3D contrast-enhanced CT obtained approximately 1 week before the study. CIPV was diagnosed when the left and right inferior PVs had the continuity behind the LA posterior wall with a common trunk connected to the LA, thus showing a protruding shape from the LA (Fig. 1). Twenty-five consecutive patients without CIPV from the same population served as controls. The Ethics Committee at our institution approved the study (approval number, 1186) and written informed consent on the ablation procedure was obtained from all patients before the procedure.

Before the ablation procedure, we performed transesophageal echocardiography in patients with a CHADS_2_ score ≥ 2, persistent AF, or a history of cerebral infarction or systemic thromboembolism to rule out an LA thrombus. All patients were administered an oral anticoagulant for at least 4 weeks before the study. In patients taking direct oral anticoagulants, the drugs were continued before the procedure, held in the morning the day of ablation, and resumed 4 h after the procedure unless major bleeding events occurred. The anticoagulants were continued for at least 3 months after the procedure.

### Cardiac catheterization

All patients underwent an AF ablation procedure in a fasting state under local anesthesia and conscious sedation with dexmedetomidine and thiamylal. Respiratory management devices, such as a nasal airway device and adaptive servo-ventilation, were used at the operator’s discretion. A 6Fr, double-decapolar, steerable catheter (BeeAT; Japan Lifeline Co., Tokyo, Japan) was inserted into the coronary sinus via the right internal jugular vein. An 8 Fr Carto SoundStar® was inserted into the right atrium via the right femoral vein, and the LA-3D geometry was created by the CartoMerge™ module, which was thereafter integrated with the 3D LA CT image. Under ICE guidance, the transseptal puncture was performed and an 8.5Fr long sheath (SL0; St. Jude Medical, St. Paul, MN, SA) and an 8.5Fr steerable sheath (Agilis; St. Jude Medical or VIZIGO, Biosense Webster) were inserted into the LA. For anticoagulation during the procedure, 3000 units of heparin were injected before and 5000 units just after the transseptal puncture, followed by repetitive administration of 1000–2000 units of heparin to maintain an activated clotting time > 300 s during the procedure. No esophageal temperature monitoring was done in this study.

### Imaging of the LA posterior wall by ICE

**Fig. 2:**
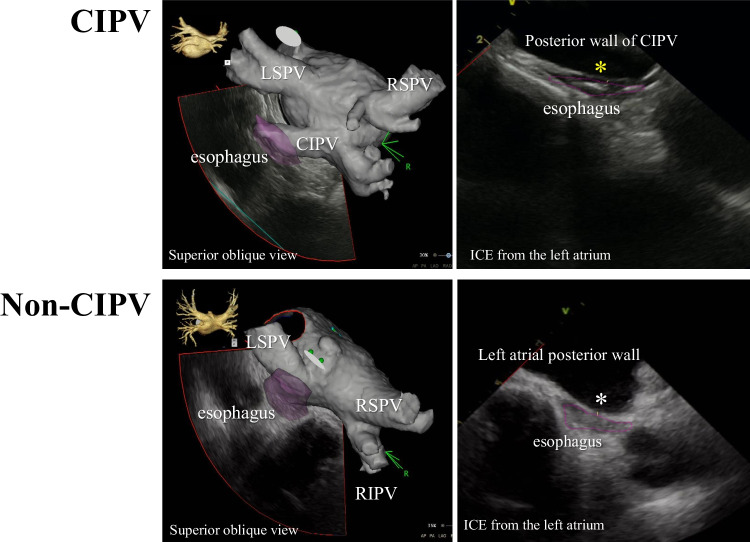
Representative intracardiac echocardiographic (ICE) images of the left atrium, pulmonary veins (PV), and esophagus in cases with confluent inferior pulmonary veins (CIPV) and with non-CIPV. The left panel show 3-dimensional (3D) computed tomographic images of the left atrium (superior oblique view) and ICE plane at the levels of CIPV and left inferior PV, revealing the thin wall of the posterior CIPV wall (yellow asterisk) and esophagus (right upper panel) and left atrial posterior wall (white asterisk) and esophagus (right lower panel). The 3D constructed esophagus is shown in purple in the left panels and the outline of the esophagus is drawn by a purple line in the right panels. LSPV, left superior pulmonary vein; RSPV, right superior pulmonary vein; RIPV, right inferior pulmonary vein

Through the SL0 sheath advanced in the LA, a Carto Soundstar® catheter was inserted into the LA [10]. The LA ICE image was created digitally with the use of a Vivid™ iq imaging console (GE Healthcare, Fairfield, CT, USA). The Soundstar® catheter was rotated and the multiple imaging planes of the LA posterior wall and esophagus were obtained. Detailed operating instructions have been reported previously [10], and for imaging we used a 6 MHz frequency among variable frequencies (4.5, 6, 8, 10, and 11.5 MHz) equipped in the Carto Soundstar®. The esophagus in the ICE imaging was characterized by two echo-lucent stripes of esophageal muscle separated by a bright echogenic lumen. Representative images of the esophagus and LA of the CIPV and non-CIPV cases are shown in Fig. 2.

### Measurement of CIPV PWT and non-CIPV LAPWT

From the LA posterior wall and esophagus images, the CIPV PWT and non-CIPV LAPWT were measured using Vivid™ iq software at the height levels of the CIPV and the left inferior PV in non-CIPV cases, respectively (Video supplement 1). The rationale for measurement at the left inferior PV level in non-CIPV cases was the distance at this level was the shortest [10].

### AF ablation procedure

The ablation index (AI)-guided circumferential PVI details have been reported previously [11]. In non-CIPV cases, circumferential PVI was performed with a Thermocool SmartTouch Surround Flow catheter (Biosense Webster) using a RF power at 40-50W except for the esophageal region (25W on the esophagus region). A target AI was ≥ 400 at the anterior wall, ≥ 360 at the posterior wall, and ≥ 260 at the esophagus. In CIPV cases, tri-circle ablation set was made: First, each superior PV was individually isolated with an AI value ≥ 400 at the anterior wall and ≥ 360 at the posterior wall under RF power (40–50W). Then, CIPV box isolation was done by making a superior and posterior transverse line over the CIPV, connecting the ablation of left and right superior PVs, and an inferior transverse line below the CIPV, which was then connected to the superior PV anterior wall ablation. Thus, the posterior wall of the CIPV was not ablated to avoid damaging the esophagus. AI targets for this box isolation was between 260 and 360, and RF power 40–50W.

The VisiTag setting included a location stability set at a minimum time of 3 s and maximum range of 2.5 mm, and force over time was set at 30% with a minimum force of 3 g. The lesion tag size had a 2-mm radius. The inter-lesion distance was < 4 mm.

In both groups a multielectrode catheter (Pentaray Nav Catheter; Biosense Webster) was inserted in each PV to confirm the disappearance of PV potentials. To confirm the PV-to-LA conduction block, pacing was done successively from the 10 pairs of the Pentaray catheter placed at the ostium of the PV at a 10-mA output with a 1-ms pulse width.

Isoproterenol (10–20 μg) was administered as a bolus to confirm PVI and to induce non-PV triggers in all patients. When non-PV triggers were detected, ablation against the triggers was attempted. The additive ablation procedures were performed at the discretion of the operators.

### Follow-up

All patients were followed up at the outpatient clinic in our hospital 1, 3, 6, 9, and 12 months after ablation. During each visit, the patients were asked if they had any symptoms suggestive of AF and underwent 12-lead ECG recording and 24-h Holter ECG monitoring. In patients with symptoms, but no ECG documentation, a mobile ECG recorder was used. AF recurrence was defined as any atrial tachyarrhythmias lasting > 30 s that occurred after a 3-month blanking period.

### Statistical analysis

Continuous variables with normal distribution are expressed as the mean ± SD and compared using a Student’s *t*-test. Continuous variables with a non-normal distribution are expressed as the median and interquartile range (IQR) and compared using a Wilcoxon test. Categorical variables are expressed as numbers and percentages and compared using Fisher’s exact or chi-square test. A *p* < 0.05 was considered statistically significant. All statistical analyses were performed with EZR (version 1.54; Saitama Medical Center, Jichi Medical University, Saitama, Japan), a graphical user interface for R (version 4.1.1; The R Foundation for Statistical Computing, Vienna, Austria) [12].

## Results

Table 1 shows the patient characteristics for CIPV (*n* = 7) and non-CIPV cases (*n* = 25). There were no differences between the two groups, except for the left ventricular ejection fraction. Of the seven CIPV cases, three had a reduced ejection fraction due to tachycardia or arrhythmia-induced cardiomyopathy. In all CIPV patients, the pre-procedural LA CT image revealed the presence of CIPV with a common trunk between the left and right PVs (type B, as defined by Yu et al. [7]; Fig. 1).

**Table 1:** Patient characteristics

	CIPV	Non-CIPV	*P* value
Number of cases	7	25	
Age (y)	65 ± 10	66 ± 12	0.85
Female, *n* (%)	1 (14.3)	6 (24.0)	1.0
Body mass index, kg/m^2^	27.1 ± 3.7	25.3 ± 3.1	0.23
CHA_2_DS_2_-VASc score	1.0 [1.0, 2.5]	1.0 [1.0, 3.0]	0.87
Type of AF, *n* (%)			0.24
Paroxysmal	3 (42.9)	18 (72.0)	
Persistent	3 (42.9)	4 (16.0)	
Long-standing	1 (14.3)	3 (12.0)	
Ejection fraction (%)	49.6 ± 16.8	61.6 ± 9.3	0.02
Serum creatinine (mg/dL)	1.0 [0.9, 1.1]	0.9 [0.8, 1.0]	0.17
Left atrium diameter (mm)	41.9 ± 5.5	42.0 ± 5.7	0.94

Values are expressed as the mean ± standard deviation or median [25^th^, 75^th^ percentile] or *n* (%). *AF* atrial fibrillation, *CIPV* confluent inferior pulmonary vein

### Measurement of CIPV PWT and non-CIPV LAWPT

**Fig. 3:**
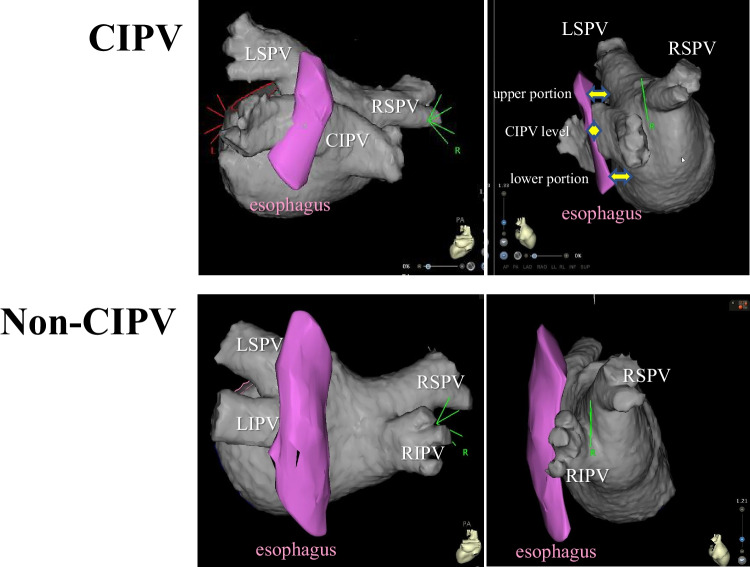
3-dimensional left atrial (gray color) and esophagus (purple color) images created by the CARTO system (left, posterior view; right, right lateral view). In all cases, the esophagus was in close contact with the posterior wall of confluent inferior pulmonary veins (CIPV) or left atrial posterior wall of non-CIPV case. Right upper panel indicates the measurement of the distances between the esophagus and the left atrium at upper and lower portions of CIPV and between the esophagus and posterior CIPV. LIPV, left inferior pulmonary vein. Other abbreviations are listed in Fig. 2

Insertion of a Carto Soundstar® into the LA and imaging of the LA posterior wall and esophagus were performed without any complications. The esophagus was in close contact with the posterior wall of all CIPV cases or the LA posterior wall of all non-CIPV cases at the middle-level height of the left inferior PV (Fig. 3). Among CIPV cases, the thinnest PWT-CIPV was 0.7 ± 0.1 mm, while the upper and lower portions of the CIPV were apart from the esophagus with mean distances of 6.7 ± 3.4 mm and 7.9 ± 2.7 mm, respectively (Fig. 3, right upper panel). Each of CIPV PWT and the distances between the LA posterior wall and esophagus at the upper and lower CIPV portions are shown in Table 2 for all cases. Among non-CIPV cases, LAPWT at the level of the left inferior PV was 2.0 ± 0.4 mm (Fig. 3, lower panel), which was significantly different from the CIPV PWT (*P* < 0.001).

**Table 2:** Distances between the left atrial posterior wall and esophagus at the upper CIPV portion, lower CIPV portion, and CIPV level in each case

Case	Upper portion	Lower portion	CIPV level
1	6.4	9.7	0.8
2	5.9	na	0.9
3	2.6	8.7	0.8
4	9.6	5.4	0.6
5	3.3	5.2	0.8
6	na	5.6	0.7
7	12.3	12.5	0.5
Mean ± SD (mm)	6.7 ± 3.4	7.9 ± 2.7	0.7 ± 0.1

*na* not available, *CIPV* confluent inferior pulmonary vein

### AI-guided PV isolation in CIPV cases

**Fig. 4:**
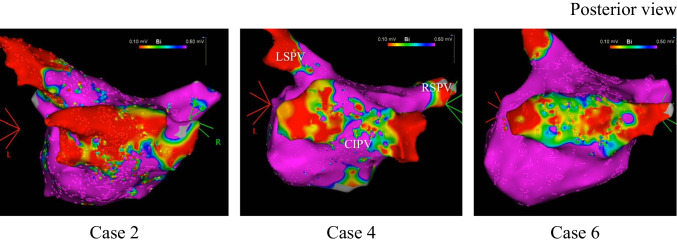
Left atrial voltage maps drawn before ablation in 3 cases with paroxysmal atrial fibrillation (posterior view). Color range indicates voltages ≥ 0.5 mV by purple color and < 0.1 mV by red color. All abbreviations are listed in Figs. 2 and 3

In three patients with paroxysmal AF, an LA voltage map was drawn during sinus rhythm or high right atrium pacing, which showed low-voltage areas with an amplitude < 0.1 mV within the CIPV (Fig. 4).

**Fig. 5:**
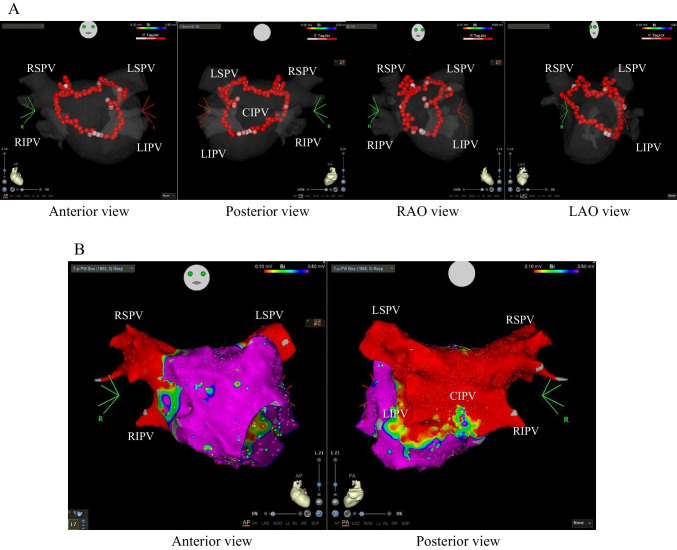
Representative case of “tri-circle” ablation. Panel **A**: Ablation index–guided ablation line (red tags) showing “tri-circle” lesions consisting of isolation of each superior pulmonary vein and box isolation of confluent inferior pulmonary veins (from left to right, anterior view; posterior view; right anterior oblique [RAO] view; left anterior oblique [LAO] view). Panel **B**: Left atrial voltage maps drawn after ablation (left, anterior view; right, posterior view). Color range indicates voltages ≥ 0.5 mV by purple color and < 0.1 mV by red color. All abbreviations are listed in Figs. 2 and 3

Individual superior PV isolation was first done for PVI, resulting in complete isolation of both left and right superior PVs. Then, CIPV box isolation was performed and resulted in complete isolation of CIPV in all cases but one (Fig. 5). In one case, box isolation was incomplete, leaving a gap at the bottom of the center of the CIPV; a double bottom line was drawn, but the gap remained. The patient had an acute recurrence of atrial fibrillation postoperatively, but has not had a recurrence in 1 year without the need for antiarrhythmic drugs. Superior vena cava isolation was performed in four cases.

In non-CIPV cases, the circumferential PV isolation under AI guidance resulted in complete PV isolation in all cases.

No major complications, such as cardiac tamponade or stroke associated with the ablation procedure, occurred.

### Follow-up in CIPV cases

All CIPV patients were followed for > 12 months. The AF-free survival rates 3, 6, and 12 months after the procedure were 100%, 100%, and 86%, respectively.

## Discussion

### Main findings

The wall thickness of CIVP compared to LAPWT of non-CIVP cases has not been described. In this study, we demonstrated for the first time the extremely thin CIPV wall thickness compared to the non-CIPV cases. This finding is clinically useful because RF application to the CIPV adjacent to the esophagus may result in excessive heating of the esophagus and may cause esophageal injury, including an atrio-esophageal fistula [13, 14]. RF application around each of the left and right superior PV ostia and CIPV box isolation is feasible for complete PV isolation. Although the incidence of CIPV may be low, considering the worldwide increase in AF ablation procedures, CIPV presence and the anatomic characteristics should be kept in mind.

### Incidence and ablation of CIPV

Because the presence of CIPV is a rare variant among AF patients undergoing catheter ablation, studies on CIPV clinical characteristics and ablation strategies have been limited. Lickfett et al. reported for the first time a common trunk variant of the right and left inferior PVs (CIPV) [15]. Lickfett et al. emphasized the usefulness of 3D imaging created based on a pre-procedural CT scan for a reliable recognition of this anomaly. By analyzing the 3D LA images obtained before the ablation procedure in 1226 consecutive AF patients, Yu et al. [7] reported 11 CIPV cases (0.9%), of whom 7 had a common ostium without a common trunk (type A, 0.6%) and the other 4 had a common ostium with a short common trunk (type B, 0.3%). Among our 986 consecutive AF patients undergoing catheter ablation, we identified 7 with CIPV and a common trunk (0.7%); the incidence was similar to the incidence reported by Yu et al. [7]. A type B CIPV with a common trunk (Fig. 1) is considered to be a true form of CIPV because the left and right inferior PVs are connected behind the LA with a common trunk connecting to the LA. The present incidence of 0.7% was low among the present AF patients, but was shown to be at a higher rate than in the previous report (0.3%) [7]. Yu et al. proposed circumferential PV isolation with “tri-circle” under the guidance of 3D-intergrated LA image for ablation. We created similar “tri-circle” lesions consisting of each superior PV and CIPV box isolation, and successfully isolated all PVs, including CIPV, without any complications.

### Wall thickness of CIPV

One of the important clinical implications of this study involved the demonstration of the thin CIPV wall thickness by ICE from the LA cavity. As shown previously, ICE provides a higher spatial resolution (maximum, 0.2–0.3 mm) compared to cardiac CT and cardiac MRI [10, 16, 17]. ICE was shown to assess the right atrial wall thickness accurately in an experimental model [18]. CartoSoundstar^Ⓡ^ produces imaging variable frequencies from 4.5 to 11.5 MHz, and provides near-field clarity within 5–7 cm of the transducer, therefore making it possible to visualize the detailed cardiac structure and other areas of interest. We previously reported the usefulness of ICE from the LA cavity in directly visualizing the esophagus and accurately measuring the LAPWT at the level of the esophagus using a 6-MHz frequency. We showed that the mean LAPWT was a minimum of 1.9 mm at the height of the left inferior PV [10]. In this study, we measured the CIPV wall thickness with ICE from the LA at the level of the esophagus, and found that the mean thickness was only 0.7 mm, whereas the mean LA PWT in non-CIPV cases was 2.0 mm at the height of the left inferior PV. The structural details of CIPV have not been reported previously; thus, we could not address the relationship between the present findings to CIPV anatomy and structure. The findings obtained from the present CIPV and non-CIPV cases suggest that CIPV includes less or no myocardial tissue or sleeve compared with the other LA area and mainly consists of vein tissue. This was further supported by the presence of broad low-voltage areas in the CIPV.

This study also showed that all CIPV cases had the upper and lower LA portions of the CIPV apart from the esophagus (mean, 6.7 and 7.9 mm, respectively; Fig. 3). Therefore, the risk of esophageal injury during ablation was minimal, allowing RF application with an AI value between 260 and 360 to be possible. We performed box isolation around the CIPV, as reported previously, without ablating inside the CIPV and individual isolation of superior PVs. This ablation strategy was feasible and reasonable in the present specific CIPV case.

Recently, a successful case of pulsed field ablation (PFA) for CIPV was reported, in which after ablating the superior PVs, CIPV and posterior wall ablation were done without any complications [19]. Future energy modalities, such as PFA, may also be used to lower risk of esophageal injury for CIPV variants.

### Study limitations

The study was conducted in a single center and included a limited number of patients. For an accurate assessment of the LA posterior wall thickness adjacent to the esophagus, the image plane needs to be perpendicular toward the posterior LA wall. We attempted to obtain such an image, but the possibility of overestimation due to an oblique projection might have occurred. Furthermore, the measurements were not blinded and therefore bias could have been introduced. We only included CIPV cases with a short common trunk (type B), but not the cases without a common trunk. Therefore, PWT was not studied in the cases without a common trunk. We did not measure the esophageal temperature during the procedure, and therefore could not show if the esophageal temperature rose or not during the tri-circle technique ablation. Furthermore, we did not perform endoscopic examination after the ablation procedure; therefore, the presence of asymptomatic esophageal lesions could not be completely excluded. Further studies are required to establish the safety of the present tri-circle ablation technique in CIPV cases. Because AF recurrence was assessed by the symptoms and periodic Holter ECG monitoring, asymptomatic AF might have been underestimated.

## Conclusions

Although the incidence of CIPV with a common trunk connecting to the LA was low (0.7%), the CIPV PWT was very thin and special care is required during ablation procedures when isolation of the inferior PVs is attempted. A “tri-circle” ablation strategy avoids ablating in the thinnest portion of the posterior wall and further studies are warranted to assess the safety.

### Supplementary Information

Below is the link to the electronic supplementary material.

Supplementary file1 (PPTX 24318 KB)

## Data Availability

The data that support the findings of this study are available from the corresponding author upon reasonable request.
