# Supplementary figures and images for: Posterior wall thickness of the confluent inferior pulmonary veins measured by left atrial intracardiac echocardiography: implications for catheter ablation
Source: J Interv Card Electrophysiol. 2023 Jul 25;67(1):193–201. doi: 10.1007/s10840-023-01613-w (PMC10770267; doi:10.1007/s10840-023-01613-w)

## Slide 1
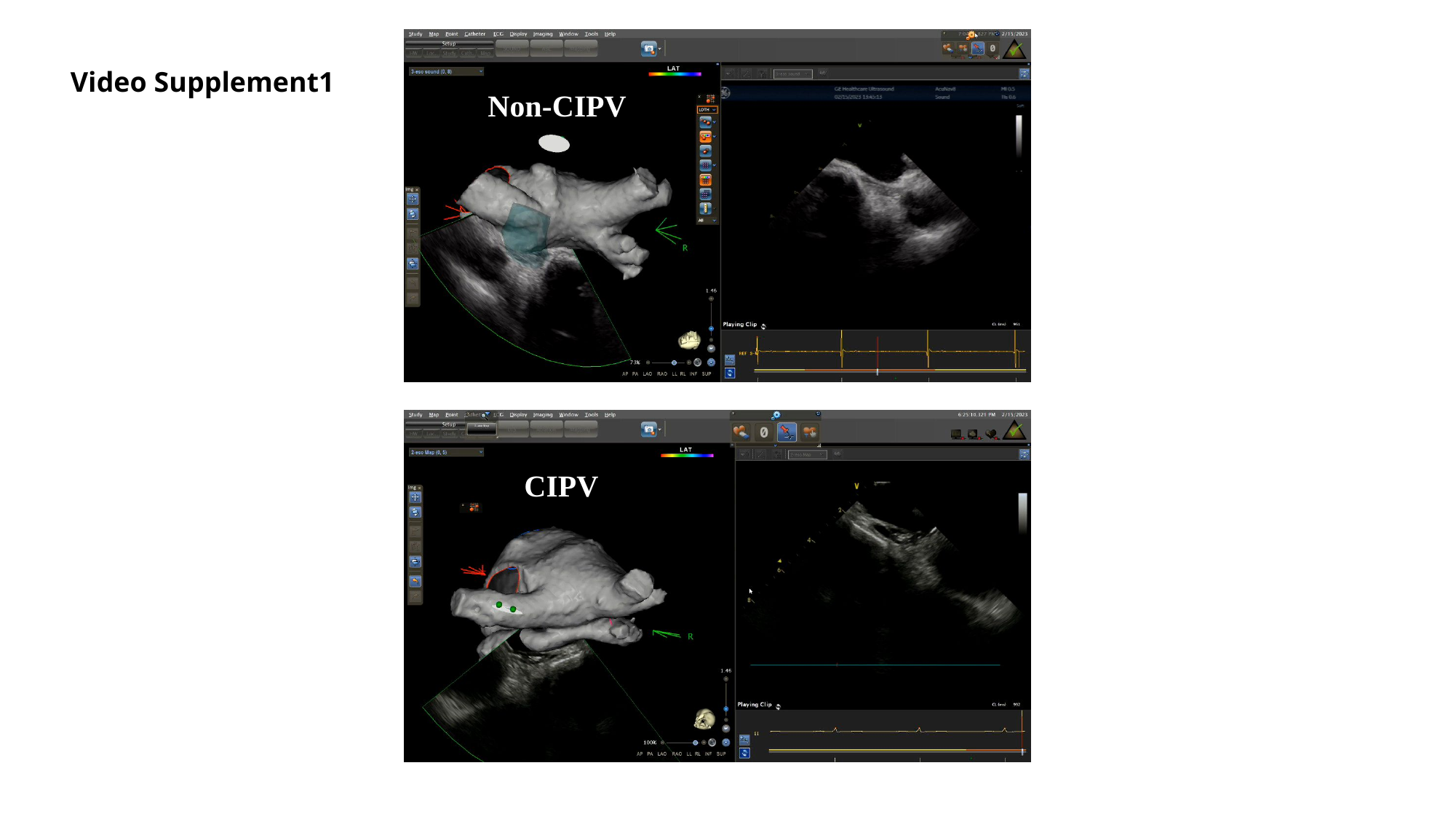

Video Supplement1
Non-CIPV
CIPV

Supplement: Supplementary file 1 — Supplementary file1 (PPTX 24318 KB) [file 10840_2023_1613_MOESM1_ESM.pptx]
